# Influences on the decision to use an osteoarthritis diagnosis in primary care: a cohort study with linked survey and electronic health record data

**DOI:** 10.1016/j.joca.2015.12.015

**Published:** 2016-05

**Authors:** K.P. Jordan, V. Tan, J.J. Edwards, Y. Chen, M. Englund, J. Hubertsson, A. Jöud, M. Porcheret, A. Turkiewicz, G. Peat

**Affiliations:** † Research Institute for Primary Care and Health Sciences, Keele University, Keele, UK; ‡ Clinical Epidemiology Unit, Orthopaedics, Department of Clinical Sciences Lund, Lund University, Lund, Sweden; § Clinical Epidemiology Research & Training Unit, Boston University School of Medicine, Boston, MA, USA; ‖ Department of Laboratory Medicine, Division of Occupational and Environmental Medicine, Lund University, Lund, Sweden

**Keywords:** Osteoarthritis, Computerized patient medical records, Primary health care

## Abstract

**Objective:**

Clinicians may record patients presenting with osteoarthritis (OA) symptoms with joint pain rather than an OA diagnosis. This may have implications for OA research studies and patient care. The objective was to assess whether older adults recorded with joint pain are similar to those with a recorded OA diagnosis.

**Method:**

A study of adults aged ≥50 years in eight United Kingdom general practices, with electronic health records linked to survey data. Patients with a recorded regional OA diagnosis were compared to those with a recorded joint pain symptom on socio-demographics, risk factors, body region, pain severity, prescribed analgesia, and potential differential diagnoses. A sub-group was compared on radiographic knee OA.

**Results:**

Thirteen thousand eight hundred and thirty-one survey responders consented to record review. One thousand four hundred and twenty-seven (10%) received an OA (*n* = 616) or joint pain (*n* = 811) code with wide practice variation. Receiving an OA diagnosis was associated with age (75+ compared to 50–64 OR 3.25; 95% Credible intervals (CrI) 2.36, 4.53), obesity (1.72; 1.22, 2.33), and pain interference (1.45; 1.09, 1.92). Analgesia management was similar. Radiographic OA was common in both groups. A quarter of those with a joint pain record received an OA diagnosis in the following 6 years.

**Conclusion:**

Recording OA diagnoses are less common than recording a joint pain symptom and associated with risk factors and severity. OA studies in primary care need to consider joint pain symptoms to understand the burden and quality of care across the spectrum of OA. Patients recorded with joint pain may represent early cases of OA with need for early intervention.

## Introduction

In the UK, the initial presentation and management of osteoarthritis (OA) most commonly occurs within primary care. The UK National Institute for Health and Care Excellence (NICE) guidance recommends application of a working diagnosis of OA in adults 45 years and older presenting with persistent joint pain, not associated with lasting morning stiffness, but excluding those with atypical features of OA^1^. EULAR guidelines recommend making a diagnosis of knee OA based on knowledge of the underlying population prevalence and the presence of patient risk factors for OA, their symptoms, and physical examination^2^. Whilst both guidelines infer that an OA diagnosis can normally be made without recourse to further investigation, there are likely to be instances of diagnostic uncertainty.

In primary care, health-related information including diagnosis is typically electronically recorded and coded. In the UK the most common system used is the Read code classification^3^ which allows health care professionals to label a presenting complaint with a symptom or disease-based Read code. Thus, OA-related symptoms may be categorised as joint pain codes rather than as an OA diagnosis. A study assessing the completeness of recorded diagnoses in primary care found a low sensitivity of 63% for OA, with a major reason being use of alternative codes, such as knee pain, by clinicians^4^. Even accounting for patients not seeking health care, there appears to be a wide discrepancy between the estimates of self-reported symptomatic OA and the prevalence of primary care recorded OA diagnosis. In the UK, it has been estimated that 53% of older adults report chronic joint pain, and 22% severe disabling pain^5^, but only 13% of older adults in the same geographical region received an OA diagnosis over a 7 year period^6^. A study in Sweden found only 63% of those with symptomatic knee OA had a recorded knee OA diagnosis within an 8 year period^7^. A prior study of ours showed there may be 10 years between recording of initial symptoms of knee pain and a recorded OA diagnosis in primary care^8^. The threshold for diagnosing and subsequently coding OA is likely to be variable, dependent on, for example, the individual practitioner's personal preference in coding, perceived reaction of the patient to receiving an OA diagnosis, or extent of uncertainty in diagnosis and wish for further confirmation such as radiographic evidence.

Understanding the spectrum of OA that is captured by a diagnosis code is important for several reasons. Primary care records are increasingly being used as a sampling frame for recruitment to trials and cohort studies, and to estimate morbidity prevalence and incidence in order to direct future health service planning^9^. Excluding older patients with joint pain symptom codes may result in selective populations in studies of OA, and under-estimated consultation prevalence and incidence of OA that has been shown in both the UK and Sweden^6^, ^10^. There is also some evidence that those recorded with a joint pain symptom rather than an OA diagnosis have different patterns and quality of care^11^.

The objective of this study was first to assess, within a cohort with linked self-report and medical record information, whether older adults with a recorded joint pain symptom in primary care have similar risk factors and pain characteristics, management, and existence of potential alternative diagnoses as those with a recorded OA diagnosis. The hypotheses tested are described in Box 1, with the underlying null hypothesis that only the recording practices of clinicians differentiates those with an OA diagnosis and those with a joint pain symptom record. The second objective was to determine the percentage of older adults recorded with a joint pain symptom who had a recorded OA diagnosis within the next 6–7 years.

## Methods

The North Staffordshire Osteoarthritis Project (NorStOP) was a longitudinal survey of all those aged 50 plus registered at 8 general practices. In the UK, most people are registered with a general practice and therefore the registers provide a convenient sampling frame for the local population. At baseline the general practitioners (GPs) at the practices excluded those with severe illness (for example, severe psychiatric or terminal illness) and questionnaires were then mailed to the remaining registered population aged 50 and over with reminders sent after 2 and 4 weeks. Further questionnaires were mailed at 3 years and 6–7 years^12^, ^13^. Self-reported survey data was linked to primary care records (with consent) with records collated from 24 months prior to the baseline survey to either the date of the 6–7 year survey or the date the participant dropped out of the study (for example, if the participant did not respond to the 3 year survey, collation of the records ended then). The primary care record follow up lasted a median of 6.4 years from the baseline survey (IQR 3.7, 6.9).

We previously identified through consensus of GPs a set of Read codes relating to non-specific joint pain (hand, hip, knee, foot) which could be used by GPs as an alternative to an OA diagnosis code for older patients presenting with likely OA^6^, ^11^ and are available from www.keele.ac.uk/mrr. Two groups were identified for this analysis from all NorStOP baseline respondents who consented to medical record review, based on their primary care consultation records for the 12 months before the baseline survey. Group 1 received an OA diagnostic code during the 12 months (OA group); group 2 received a joint pain symptom code but not an OA diagnostic code during those 12 months (joint pain group). Respondents who received both an OA diagnostic code and a joint pain code were included in the OA group. Both groups included patients with ongoing problems and those consulting with new problems. The index date was the date of the recorded OA/joint pain code nearest to the baseline survey within this 12 month time period. Respondents for whom a body region (knee, hip, hand/wrist, foot/ankle) at the index date consultation could not be allocated, either through the code given or recorded in the free text of the consultation, were excluded in order to allow comparison by individual site.

In order to address the hypotheses stated in Box 1, information on pain medication prescribing and differential diagnoses were extracted from the medical records, information on socio-demographic risk factors, extent of pain, pain interference, body mass index (BMI), and anxiety and depression were identified from the baseline survey, and radiographic information from a subset of respondents undergoing radiographs.

The OA and joint pain groups were first compared on socio-demographic risk factors (age, gender, socioeconomic status) and other known or proposed risk factors (BMI, depression or anxiety) measured in the baseline survey^2^, ^14^, ^15^. They were also compared on body region consulted for at index consultation, extent of and interference from pain at time of baseline survey, and analgesia management (at time of index consultation).

Socio-economic status was based on reported current or last job^16^, and categorised into low social class (lower supervisory, lower technical, semi-routine or routine occupations), high social class (managerial/professional, intermediate occupations/self-employed) and unknown based on the highest social class of the individual or their spouse.

Extent of pain was measured by self-reported number of sites of pain over the past 12 months (count of knee, hip, hand and foot) in the baseline survey. This was based on four questions, one for each site, with answer options of yes or no. For example, the question relating to foot pain was “Have you had pain in the last year in and around the foot?”. Pain interference was defined using the Short Form-12 item “During the past 4 weeks, how much did pain interfere with your normal work (including both work outside the home and housework)”. Respondents who responded “moderately”, “quite a bit” or “extremely” were rated as having pain interference, whilst those who responded “not at all” or “a little bit” were regarded as not having pain interference^13^, ^17^, ^18^, ^19^.

BMI was determined using self-reported height and weight and categorised as normal/underweight (BMI ≤ 25 kg/m^2^), overweight (>25–30), and obese (>30). Anxiety and depression in the baseline survey were classified using the Hospital Anxiety and Depression Scale (HADS)^20^. The HADS contains 14 items from which a score (range 0–21) for each of anxiety and depression can be determined. Scores of 8 or more on either domain indicate possible or probable anxiety or depression.

Analgesia prescribed at time of the index consultation were identified through the primary care records using a hierarchical categorisation derived by Bedson and colleagues^21^. This splits analgesics into basic analgesics (for example, paracetamol), weak or moderate opioids (for example, codeine 8 mg–15 mg), strong opioids (for example, codeine 30 mg, morphine and oxycodone) and non-steroidal anti-inflammatory drugs (NSAIDs). Pain medication was deemed as being related to an OA or joint pain consultation if it was prescribed on the same day as the index consultation, or within 14 days of that consultation. This was to allow for patients to take up a ‘delayed’ prescription for analgesics that the GP offered should the condition not improve.

Recording of a potential differential diagnosis in the primary care records was also assessed.

Differential diagnoses were determined by consensus of 3 GPs and identified in the primary care records for the 12 months preceding and 12 months following the date of the index consultation. These included inflammatory musculoskeletal diagnoses such as rheumatoid arthritis, joint specific diagnoses such as bursitis and enthesopathy, and generalised conditions such as fibromyalgia (Supplementary table).

A subgroup of the NorStOP respondents who reported knee pain also had plain radiographs taken of their knee with three views to capture both tibiofemoral and patellofemoral OA^22^, ^23^, ^24^, ^25^. Mild knee OA was defined as a Kellgren and Lawrence (K&L) score of 2 in the posteroanterior or skyline view, or a score of 1 or 2 on posterior or lateral osteophytes. Moderate/severe knee OA was defined as a K&L score of ≥3 in the posteroanterior or skyline view, or a score of 3 on posterior or lateral osteophytes^26^. A single reader scored all films and was blinded to all questionnaire data. Intraobserver and interobserver (with a second reader) agreement of posteroanterior K&L score, skyline K&L score and lateral osteophytes were assessed on 100 knees. Unweighted kappas for intraobserver reliability were between 0.81 and 0.98; interobserver kappas were between 0.49 and 0.76^27^. The proportion of these respondents classified as having no, mild or moderate/severe radiographic knee OA in their most problematic knee was compared between the knee OA and joint pain groups.

### Statistical analysis

Multilevel logistic regression models (patients clustered within practices) were used for the analysis. First the variance components model (i.e., with no explanatory variables included) was derived to assess the amount of variation in use of an OA diagnosis code that was at the practice level compared to that between respondents^28^. Then associations with a recorded OA diagnosis were determined first unadjusted, and then fully adjusted with all the socio-demographic and other risk factors, pain extent and interference, and analgesic prescription included in the model. Results are reported as odds ratios (OR) with 95% credible intervals (95% CrI) obtained through Markov Chain Monte Carlo (MCMC) methods. The analysis was repeated in three subgroups: (1) those with no recorded OA or joint pain consultation in the 12 months prior to their index consultation (new episode consulters) to assess whether the associations with an OA diagnosis persisted or altered in new consulters, (2) those consulting for a problem related to the knee, and (3) those consulting for a problem related to the hip. There were too few patients consulting for hand/wrist or foot/ankle to allow further exploration of these sites. In the subgroup with radiographs we compared patients receiving a knee OA diagnosis to those with a knee joint pain record on presence and severity of knee radiographic evidence of OA, unadjusted and adjusting for age and gender.

We also determined the percentage of those in the joint pain group who had a pre-existing OA diagnosis in their primary care records in the period 12–24 months before the baseline survey, and the percentage recorded with an OA diagnosis, inflammatory musculoskeletal condition, joint specific soft tissue diagnosis, or fibromyalgia after the survey during the follow up period.

Analyses were performed using runmlwin^29^, MLwiN 2.29^30^, ^31^, and Stata 13.1.

## Results

Twenty six thousand six hundred and twenty-five people were mailed a baseline questionnaire. Of these, 186 were excluded due to death or departure from their practice, 240 had an incorrect address, 22 were subsequently found to be ineligible, and 48 were excluded due to severe ill-health. Of the remainder,18,497 (71%) responded. 13,831 (75%) of these consented to medical record review. 1741 (13%) had received an OA diagnosis or joint pain code in the 12 months prior to the baseline survey, of which 1427 were able to be allocated to a body region. Of these 1427, 616 (43%) had a recorded OA diagnosis and 811 had a joint pain symptom recorded. However there was variation by practice. One practice recorded only 19% with an OA diagnosis, whilst another practice recorded 70% with an OA diagnosis. (Table I) Prior to inclusion of explanatory variables in the multilevel model, 16% of the variation in coding was at the practice rather than respondent level.

Increasing age was strongly associated with receiving an OA diagnosis with those aged 75 and over having more than 3 times the odds of a recorded OA diagnosis than those aged 50–64 (adjusted OR 3.25; 95% CrI 2.36, 4.53). Obesity (OR 1.72; 95% CrI 1.22, 2.33) was also associated with a recorded OA diagnosis. There were no statistically significant associations with gender, social class, or anxiety/depression (Table II).

Reporting interfering pain was associated with a recorded OA diagnosis (adjusted OR 1.45; 95% CrI 1.09, 1.92), however 62% of those with a joint pain record reported pain interference and the number of self-reported pain sites was statistically significantly associated with a recorded OA diagnosis in the unadjusted analysis only. Those consulting with a hip problem (OR 0.50; 95% CrI 0.36, 0.67) or foot/ankle problem (OR 0.26; 95% CrI 0.17, 0.38) were less likely to have a recorded OA diagnosis than someone presenting with a knee problem. The prescription of analgesia and strength of analgesia prescribed were similar between groups.

The associations generally persisted in the subgroups consulting with a new episode and in the subgroups with a knee and hip problem (Table III, Table IV) although the associations with obesity (OR 1.67; 95% CrI 0.97, 2.64) and pain interference (OR 1.35; 95% CrI 0.82, 1.99) in those with a new episode were weaker and became statistically non-significant. The relationship of recorded OA diagnosis with older age was stronger in those with a knee problem (age 75+: OR 8.97; 95% CrI 5.32, 14.78).

32% of all those with recorded joint pain and 26% of all those with an OA diagnosis record had a possible differential diagnosis in their records (chi-squared test, *P* = 0.014, Supplementary table). The main difference between the groups was in the presence of another or unspecified arthropathy code (11% of the joint pain group compared to 5% of the OA group). Those with a hand/wrist problem were most likely to have a differential diagnosis (42% of joint pain group and 27% of OA group) and those with a hip problem were least likely (25% of joint pain group and 21% of OA group).

In the 124 patients with a knee problem for whom plain knee radiographs were available from the nested sub-study, the presence of radiographic features of OA was associated, albeit not statistically-significantly, with an OA diagnosis (Table V). Those with moderate or severe radiographic OA had nearly 3 times the odds of an OA diagnosis compared to those without radiographic OA (unadjusted OR 2.82; 95% CrI 0.93, 6.78). However, 52% of the joint pain group had radiographic evidence of moderate or severe OA.

Of the 811 patients in the joint pain group, 53 (7%) had a prior recorded OA diagnosis (any body region) in the period 12–24 months prior to the baseline survey. In the median 6 years after the baseline survey, 203 (25%) of the 811 patients in the joint pain group received an OA diagnosis. Twenty-five (3%) of the joint pain patients had a recorded long term inflammatory musculoskeletal condition such as rheumatoid arthritis during this same follow-up period, 21 (3%) had a recorded specific soft tissue diagnosis such as bursitis, and three had fibromyalgia recorded. In the 447 joint pain patients with a new consulting episode (no joint pain or OA record in 12 months prior to index date), 111 (25%) had a recorded OA diagnosis after the baseline survey.

## Discussion

Variation exists in the relative recording of joint pain symptoms and OA diagnosis in older adults presenting to primary care. Older age, obesity and interference of pain increased the likelihood of being recorded with an OA diagnosis although these risk factors were also common in those with a joint pain record. However, those with foot and ankle problems were less likely to have a recorded OA diagnosis than those with knee problems. Prescription management was not associated with an OA diagnosis.

Our a priori hypotheses to test whether older patients with an OA diagnosis record and those with a joint pain record are a similar group generally did not hold, except for pain medication management, which suggests that there are some distinct differences between those who are recorded with an OA diagnosis and those recorded as having a joint pain symptom. It appears that GPs may often reserve the diagnosis for those who reach a threshold in severity of symptoms, fit the risk factor profile (older age, obese) typically associated with OA, and thus in whom they feel more confident in making a positive diagnosis of OA. However, whilst there was an elevated likelihood of an OA diagnosis in those with moderate or severe radiographic OA, half of those in the joint pain group also had moderate or severe radiographic OA, and over half reported interfering pain, suggesting many of those recorded with a joint pain symptom do have severe problems.

Those presenting with a hip or foot/ankle problem were less likely to receive an OA code than those presenting with a knee problem. Pain in the foot and ankle in particular may be attributed to many conditions other than OA so this may reflect an appropriate level of caution by GPs. Those with a hand/wrist joint pain record were more likely to have a potential differential diagnosis. Joint pain codes for hand/wrist generally do not relate to individual joints in the hand/wrist but to the whole hand or wrist, for example, “hand pain”, therefore it was not possible to determine likelihood of OA based on the individual joints affected within the hand. A similar limitation occurred for foot/ankle pain. Further research needs to assess whether a foot or hand pain code given to older adults is likely to reflect OA.

A quarter of those recorded as a joint pain symptom did receive an OA diagnosis over the following 6–7 years, and only 6% received an inflammatory musculoskeletal or specific soft tissue diagnosis. This suggests that although GPs may be disinclined to offer a definitive OA diagnosis early on, they may be happy to do so after a ‘watch and wait’ policy which may allow assessment of therapeutic efficacy, other investigations to be undertaken, or symptoms to worsen to aid or help justify diagnosis. Some clinicians may be reluctant to label younger patients with a diagnosis of chronic disease. Whilst it is recognised that applying a label to a chronic condition such as OA may help to ease the anxiety that compelled the patient to seek medical intervention in the first instance, it may also alter the patient's perception of the problem from one of ongoing pain, to a label of a chronic, degenerative, incurable condition^32^, ^33^. Moreover, it has been argued that patients seek treatment for pain and not OA, with effective management reliant on presenting symptoms rather than diagnostic label. As such, it has been proposed that joint pain in adults be viewed as a regional pain syndrome much like ‘low-back pain’ rather than considered according to its disease-specific cause^33^, allowing treatment to be tailored to meet the patient's needs rather than diagnosis. The similarity in pain medication management between the two groups shown in this study suggests that recording a symptom rather than an OA diagnosis does not influence the choice of analgesia. However, given there is evidence that the quality of care may differ between those with an OA or joint pain label^11^, further research is needed to assess whether the choice of label affects recommended non-pharmacological management such as exercise, weight loss advice and referral to physiotherapy. If so, there may be missed opportunities in the management of OA, particularly early intervention at the start of health care use for joint pain in older adults, in those with initially less severe symptoms.

The choice of recorded label also has public health implications. The electronic patient record and consequent collation of primary care consultation data has created opportunities for epidemiological studies to inform health care need and service provision. Studies focussing only on those with a recorded OA diagnosis will be identifying the older, more severe patients, and may underestimate the scale of the burden of OA in primary care, and ignore the group who may have fewer of the recognised risk factors but who may progress to a later OA diagnosis. Approaches to measure and improve quality of care could also be misguided if based only on those with a recorded diagnosis of OA.

The study was limited to one area of the UK, albeit with similar musculoskeletal prevalence figures to those shown nationally, including for OA and arthralgia^34^. Whilst Read codes are the most common method of classification in general practice in the UK, they are not used elsewhere. However, we have previously matched Read codes for OA and joint pain to the International Classification of Diseases (ICD-10) and obtained very similar consultation prevalence figures for OA and for joint pain in patients aged 45 and over between the UK and Sweden^6^. This suggests that the issues highlighted here may also be of relevance outside of the UK. One practice which showed high recording of OA had been trained to preferentially record diagnoses due to their inclusion in another research database. Excluding this practice did not change the associations shown. The potential differential diagnoses identified may relate to a different problem than the OA or joint pain record and so it is not known how many of these represent true differential diagnoses. Subsequent diagnoses were also not matched to the same body region as the index joint pain consultation. It is possible that the recording of an OA diagnosis may also be associated with referral to secondary care but we were unable to explore that in this study. Whilst the GP may have recorded the consultation with a joint pain symptom, it is possible they informed the patient it was OA. We only considered a period of 12 months prior to the survey so that self-reported information was not too long after the consultation. There will be other respondents to the survey who had consulted and been recorded with an OA diagnosis or joint pain symptom more than 12 months before the survey but not in the 12 month period considered here.

Recording an OA diagnosis in elderly patients in primary care is less common than recording a non-specific joint pain symptom, and is associated with known risk factors and severity of the problem. However, those not given an OA diagnosis had high levels of interfering pain and radiographic OA. A quarter later received an OA diagnosis. This suggests that epidemiological studies of OA in primary care may need to consider joint pain symptoms as well as diagnosed OA to understand the true burden and quality of care across the full spectrum of OA. There is a need to reduce GP variation in diagnosing OA, and to understand whether there are consequences for the patient with OA symptoms of not being diagnosed with OA in terms of missed early opportunities in management of OA.

## Contributions

KJ, VT, JE, MP and GP conceived the study. All authors contributed to the design of the study. YC and KJ performed the analysis. KJ led the draft of the manuscript. All authors interpreted the findings, revised the manuscript and approved the final version.

## Competing interests

None.

## Funding

The NorStOP and CAS-K studies were funded by a Programme Grant awarded by the Medical Research Council, UK (Grant Code: G9900220) and by funding secured from the North Staffordshire Primary Care R&D Consortium for NHS service support costs. The CAS-K study was additionally funded by a Programme Grant from Arthritis Research UK (Grant Code: 18175). The funders had no role in the design, collection of the data, analysis, interpretation of the study or writing of the manuscript.

## Figures and Tables

**Table I:** Number of patients with a recorded OA diagnosis or joint pain symptom by practice

Practice	OA *n* (%)	Joint pain *n*	Total
1	176 (70)	77	253
2	95 (54)	81	176
3	86 (44)	110	196
4	50 (37)	84	134
5	91 (37)	156	247
6	44 (34)	85	129
7	50 (31)	113	163
8	24 (19)	105	129
Total	616 (43)	811	1427

**Table II:** Comparison between those with a recorded OA diagnosis and those with a recorded non-specific joint pain symptom

	OA *n* (%)	Joint pain *n*	OR (95% CrI)∗	OR (95% CrI)†
Total	616 (43)	811		
Male	229 (37)	387	1	1.00
Female	387 (48)	424	1.21 (0.96, 1.50)	1.14 (0.87, 1.45)
Age				
50–64	201 (34)	389	1.00	1.00
65–74	216 (45)	262	1.75 (1.32, 2.24)	1.78 (1.34, 2.34)
75+	199 (55)	160	2.63 (1.98, 3.49)	3.25 (2.36, 4.53)
Social class				
Low	290 (43)	390	1.00	1.00
High	300 (44)	379	1.04 (0.81, 1.31)	1.02 (0.79, 1.27)
Unknown	26 (38)	42	0.71 (0.39, 1.16)	0.57 (0.29, 1.02)
BMI group				
Normal	173 (39)	270	1.00	1.00
Overweight	261 (43)	341	1.29 (0.98, 1.65)	1.25 (0.94, 1.64)
Obese	156 (49)	165	1.61 (1.18, 2.17)	1.72 (1.22, 2.33)
Unknown	26 (43)	35	1.23 (0.66, 2.14)	1.22 (0.57, 2.18)
Neither depressed nor anxious	289 (42)	395	1.00	1.00
Depressed or anxious	312 (44)	395	1.01 (0.81, 1.26)	0.90 (0.68, 1.17)
Recorded site				
Knee	367 (50)	361	1.00	1.00
Hip	119 (37)	206	0.52 (0.39, 0.69)	0.50 (0.36, 0.67)
Hand/wrist	75 (47)	86	0.87 (0.59, 1.24)	1.03 (0.69, 1.48)
Foot/ankle	55 (26)	74	0.29 (0.19, 0.40)	0.26 (0.17, 0.38)
No. of pain sites, median (IQR)‡	2 (2–3)	2 (1–3)	1.11 (1.02, 1.22)	1.04 (0.92, 1.15)
No pain interference	155 (34)	302	1.00	1.00
Pain interference	444 (48)	489	1.70 (1.33, 2.14)	1.45 (1.09, 1.92)
No analgesic	248 (40)	375	1.00	1.00
Any analgesic∗∗	368 (46)	436	1.22 (0.97, 1.53)	–
Basic	77 (44)	100	1.17 (0.81, 1.64)	0.93 (0.62, 1.37)
Weak/moderate opioid	83 (48)	91	1.41 (0.97, 1.94)	1.04 (0.68, 1.49)
NSAID	43 (41)	63	1.01 (0.62, 1.55)	0.89 (0.53, 1.40)
Strong opioid	165 (48)	182	1.21 (0.90, 1.59)	1.07 (0.79, 1.43)

∗ Multilevel logistic regression, level 1 (patient), level 2 (practice), unadjusted.

† Multilevel logistic regression, level 1 (patient), level 2 (practice), adjusted for other listed variables.

‡ Self-reported in baseline survey (knee, hip, hand, foot).

∗∗ Prescribed on day of index consultation or in following 0–14 days.

**Table III:** Comparison between those with a recorded OA diagnosis and those with a recorded non-specific joint pain symptom in new episode consulters

	OA *n* (%)	Joint pain *n* (%)	OR (95% CrI)∗	OR (95% CrI)†
Total	254 (36)	447	–	–
Male	95 (35)	173	1.00	1.00
Female	159 (37)	274	1.16 (0.82, 1.58)	1.19 (0.81, 1.71)
Age				
50–64	90 (30)	213	1.00	1.00
65–74	90 (37)	153	1.41 (0.97, 1.97)	1.59 (1.07, 2.37)
75+	74 (48)	81	2.04 (1.30, 2.96)	2.56 (1.52, 4.03)
Social class				
Low	117 (35)	216	1.00	1.00
High	130 (39)	206	1.19 (0.85, 1.66)	1.14 (0.77, 1.67)
Unknown	7 (22)	25	0.52 (0.19, 1.09)	0.39 (0.11, 0.97)
BMI group				
Normal	80 (34)	155	1.00	1.00
Overweight	101 (35)	186	1.09 (0.73, 1.58)	1.01 (0.65, 1.50)
Obese	60 (42)	83	1.60 (0.98, 2.49)	1.67 (0.97, 2.64)
Unknown	13 (36)	23	1.16 (0.49, 2.22)	1.08 (0.41, 2.38)
Neither depressed nor anxious	133 (37)	222	1.00	1.00
Depressed or anxious	114 (35)	213	0.87 (0.61, 1.16)	0.79 (0.52, 1.15)
Recorded site				
Knee	144 (42)	203	1.00	1.00
Hip	36 (27)	99	0.39 (0.23, 0.62)	0.38 (0.22, 0.60)
Hand/wrist	39 (43)	52	1.09 (0.65, 1.68)	1.24 (0.69, 2.01)
Foot/ankle	35 (27)	93	0.43 (0.26, 0.67)	0.38 (0.21, 0.64)
No. of pain sites, median (IQR)‡	2 (1–3)	2 (1–3)	1.06 (0.93, 1.22)	1.05 (0.87, 1.25)
No pain interference	86 (31)	190	1.00	1.00
Pain interference	162 (40)	246	1.33 (0.94, 1.59)	1.35 (0.82, 1.99)
No analgesic	99 (33)	202	1.00	1.00
Any analgesic∗∗	151 (38)	245	1.22 (0.86, 1.72)	–
Basic	44 (40)	65	1.43 (0.85, 2.27)	1.31 (0.71, 2.13)
Weak/moderate opioid	34 (38)	55	1.29 (0.74, 2.06)	1.07 (0.58, 1.82)
NSAID	15 (35)	28	0.98 (0.45, 1.83)	0.91 (0.36, 1.83)
Strong opioid	62 (39)	97	1.16 (0.73, 1.76)	0.97 (0.58, 1.53)

∗ Multilevel logistic regression, level 1 (patient), level 2 (practice), unadjusted.

† Multilevel logistic regression, level 1 (patient), level 2 (practice), adjusted for other listed variables.

‡ Self-reported in baseline survey (knee, hip, hand, foot).

∗∗ Prescribed on day of index consultation or in following 0–14 days.

**Table IV:** Comparison between those with a recorded OA diagnosis and those with a recorded non-specific joint pain symptom in a) those with a knee problem and b) those with a hip problem

		Knee			Hip	
	OA (%)	Joint pain	OR (95% CrI)∗	OA (%)	Joint pain	OR (95% CrI)∗
Total	367 (50)	361	–	119 (37)	206	–
Male	140 (48)	152	1.00	49 (38)	81	1.00
Female	227 (52)	209	1.15 (0.76, 1.63)	70 (36)	125	0.87 (0.47, 1.46)
Age						
50–64	100 (35)	186	1.00	36 (29)	88	1.00
65–74	136 (53)	121	2.81 (1.87, 4.03)	48 (44)	62	1.97 (0.93, 3.62)
75+	131 (71)	54	8.97 (5.32, 14.78)	35 (38)	56	1.69 (0.78, 3.24)
Social class						
Low	169 (49)	175	1.00	59 (38)	98	1.00
High	186 (53)	166	1.22 (0.84, 1.68)	52 (35)	95	0.73 (0.39, 1.23)
Unknown	12 (38)	20	0.39 (0.13, 0.85)	8 (38)	13	0.56 (0.13, 1.49)
BMI group						
Normal	94 (49)	97	1.00	31 (26)	87	1.00
Overweight	146 (47)	167	1.06 (0.68, 1.58)	58 (43)	78	2.38 (1.17, 4.23)
Obese	110 (58)	80	1.92 (1.13, 2.99)	24 (41)	34	2.30 (0.98, 4.72)
Unknown	17 (50)	17	1.55 (0.55, 3.47)	6 (46)	7	2.50 (0.52, 7.76)
Neither depressed nor anxious	177 (48)	191	1.00	52 (39)	82	1.00
Depressed or anxious	184 (53)	164	0.96 (0.63, 1.36)	62 (34)	118	0.78 (0.41, 1.35)
No. of pain sites, median (IQR)†	2 (2–3)	2 (1–3)	1.11 (0.93, 1.30)	3 (2–4)	3 (2–4)	0.90 (0.71, 1.14)
No pain interference	90 (39)	142	1.00	21 (24)	65	1.00
Pain interference	269 (56)	209	1.47 (0.96, 2.21)	94 (41)	138	2.42 (1.09, 4.71)
No analgesic	149 (49)	157	1.00	43 (35)	80	1.00
Any analgesic‡	218 (52)	204	–	76 (38)	126	–
Basic	40 (43)	53	0.64 (0.34, 1.10)	16 (42)	22	1.19 (0.42, 2.61)
Weak/moderate opioid	52 (55)	43	0.83 (0.46, 1.43)	18 (38)	30	1.19 (0.48, 2.53)
NSAID	26 (52)	24	0.77 (0.35, 1.51)	13 (31)	29	1.00 (0.37, 2.19)
Strong opioid	100 (54)	84	1.07 (0.69, 1.63)	29 (39)	45	1.17 (0.56, 2.23)

∗ Multilevel logistic regression, level 1 (patient), level 2 (practice), adjusted for other listed variables.

† Self-reported in baseline survey (knee, hip, hand, foot).

‡ Prescribed on day of index consultation or in following 0–14 days.

**Table V:** Radiographic evidence of OA in those with a knee OA diagnosis or recorded knee joint pain symptom

Radiographic finding	OA *n* (%)	Joint pain *n* (%)	OR (95% CrI)∗
No OA	9 (14)	15 (26)	1.00
Any OA	57 (86)	43 (74)	2.87 (0.95, 6.37)
Mild OA	14 (21)	13 (22)	2.24 (0.62, 5.92)
Moderate/Severe OA	43 (65)	30 (52)	2.82 (0.93, 6.78)

∗ Multilevel logistic regression, level 1 (patient), level 2 (practice), unadjusted.

## Box 1 Hypotheses tested to assess the assumption that older patients with a recorded OA diagnosis and those with a joint pain symptom record are a homogenous group

People aged 50 and over with a non-specific joint pain symptom record differ from those with a recorded OA diagnosis only by recording practice by clinicians and not by:

i) distribution of perceived risk factors (age, gender, socio-economic status, obesity, anxiety/depression)
  • The alternative hypothesis is that an OA diagnosis is more likely to be given to patients who have recognised risk factors for OA.
ii) reporting of more severe symptoms and radiographic evidence
  • The alternative hypothesis is that an OA diagnosis is given to patients with more severe symptoms and radiographic features.
iii) recorded body region (knee, hip, hand/wrist, foot/ankle)
  • The alternative hypothesis is that an OA diagnosis is less likely for those presenting with foot/ankle symptoms where other diagnoses (for example, tendinitis, plantar fasciitis, gout) may be as likely to be the cause of the problem.
iv) prescription management
  • The alternative hypothesis is that an OA diagnosis is more likely to be given if a patient receives stronger analgesia.
v) levels of recording of differential diagnoses
  • The alternative hypothesis is that a joint pain diagnosis relates to an underlying non-OA condition.
