# Supplementary material for: Influences on the decision to use an osteoarthritis diagnosis in primary care: a cohort study with linked survey and electronic health record data
Source: Osteoarthritis Cartilage. 2016 May;24(5):786–93. doi: 10.1016/j.joca.2015.12.015 (PMC4850243; doi:10.1016/j.joca.2015.12.015)
Supplement: Supplementary file 1 [file mmc1.docx]

Supplementary Table - Differential diagnoses^a^ in those with a recorded OA diagnosis or recorded non-specific joint pain symptom

|  | OA  *n* (%) | Joint pain  *n* (%) |
| --- | --- | --- |
| Any differential diagnosis | 159 (26) | 258 (32) |
| Generalised inflammatory | 10 (2) | 24 (3) |
| Psoriatic arthritis | 0 (0) | 1 (<1) |
| Sicca syndrome | 0 (0) | 1 (<1) |
| Reactive arthropathy | 0 (0) | 2 (<1) |
| Crystal arthropathies | 2 (<1) | 6 (<1) |
| Rheumatoid arthritis | 6 (1) | 11 (1) |
| Inflammatory spondylopathies | 2 (<1) | 3 (<1) |
| Generalised other | 41 (7) | 111 (14) |
| Allergic arthritis | 1 (<1) | 0 (0) |
| Polyarthritis | 5 (<1) | 16 (2) |
| Other or unspecified arthropathy | 32 (5) | 89 (11) |
| Fibromyalgia | 1 (<1) | 3 (<1) |
| Osteomyelitis, periostitis and other infections affecting bone | 2 (<1) | 3 (<1) |
| Joint specific inflammatory | 19 (3) | 21 (3) |
| Synovitis tenosynovitis | 17 (3) | 19 (2) |
| Specific bursitides | 1 (<1) | 1 (<1) |
| Bursitis NOS | 1 <1) | 1 (<1) |
| Joint specific other | 89 (14) | 102 (13) |
| Pyogenic arthritis | 1 (<1) | 0 (0) |
| Unspecified monoarthritis | 1 (<1) | 1 (<1) |
| Internal derangement | 2 (<1) | 5 (<1) |
| Other derangement | 0 (0) | 1 (<1) |
| Enthesopathy hip | 10 (2) | 17 (2) |
| Enthesopathy knee | 4 (<1) | 6 (<1) |
| Enthesopathy ankle | 38 (6) | 43 (5) |
| Enthesopathy NOS | 14 (2) | 9 (1) |
| Ganglion | 19 (3) | 20 (2) |
| Rupture | 0 (0) | 0 (0.0) |

^a^ diagnosis closest to index date if more than one diagnosis recorded

OA: *n* = 616; joint pain: *n* = 811

NOS = Not otherwise specified
